# Probable Carcinomatous Tumors Encroaching upon the Alimentary Tract

**Published:** 1882-01

**Authors:** E. P. Bowman


### IX.—PROBABLE CARCINOMATOUS TUMORS ENCROACHING UPON THE ALIMENTARY TRACT.

---

REPORTED BY DR. E. P. BOWMAN.

---

A mare, 18 years old, owned in Boston, and a roadster of previously good repute, was observed to have less than her usual spirit when taken from her winter quarters last spring. Her appetite also was poor, and the bowels were constipated. Not much attention, however, was paid to the matter until about three weeks ago, when I was called to the case. On examination, I found the pulse, temperature and respiration normal, but there was a slight yellow tinge to the mucous membrane of the mouth and tongue, and conjunctiva of the eye. The upper and lower molars, also, were unusually sharp, so much so as to cut the tongue and cheeks. Judging that this condition might have caused the symptoms alluded to, I dressed the teeth. She was then sent into the pasture field, where she fed heartily for a day or two. On August 24, I was again requested to see her, and found the following conditions: Pulse 60, temperature 100, respiration 20; vision imperfect; the yellowish tinging of the conjunctivæ more marked than at my first visit; decided nervousness, and some thirst. I ordered bran-mashes with laxatives, following them up with an active purge. On August 28, pulse, temperature and respiration were normal, and the conjunctivæ were clear; appetite poor, and a tonic ordered. On August 30, I found the animal lying down, and unable to rise. She was bathed in a profuse perspiration. Foreseeing a rapidly fatal termination, I recommended that she be put out of the way as soon as possible.

At post-mortem examination, the following facts were revealed:

After exposing the abdominal cavity, about three gallons of a servous fluid escaped. On inspecting the intestines, I expected to find that foreign bodies had given rise to the trouble, as in my experience carpet tacks, shingle-nails, or even large intestinal calculi produced symptoms somewhat analagous to those described in this case. I found, however, that the whole attack was mainly attributed to a stricture of the gut, caused by two tumors that pressed upon and partially occluded it. A microscopic examination was made of the growths, and, indeed, the autopsy was conducted rather hastily, owing to the heat of the weather. In my opinion, the disease was cancerous, and I judge so by the hardness of the growths and their known proclivity to attack these parts.

LYNN, Mass.

[NOTE.—If specimens similar to the above-described will be sent, preserved first one week in Müller's fluid:

℞

| | |
|---|---|
| Potassium Bichromate, | 2 parts. |
| Sodium Sulphate, | 1 part. |
| Aquæ, | 100 parts. |

Mx.

And then, after washing in water in 95 per cent. alcohol, to the laboratory of the Columbia Veterinary College, 217 East 34th Street, they will be examined, and a report of the examination and of the case inserted in the columns of this journal.—ED.]

